# Role of Lectin-like Oxidized LDL Receptor-1 and Syncytiotrophoblast Extracellular Vesicles in the Vascular Reactivity of Mouse Uterine Arteries During Pregnancy

**DOI:** 10.1038/s41598-020-63205-2

**Published:** 2020-04-08

**Authors:** Floor Spaans, Anita Quon, Raven Kirschenman, Jude S. Morton, Tatsuya Sawamura, Dionne S. Tannetta, Ian L. Sargent, Sandra T. Davidge

**Affiliations:** 1 Department of Obstetrics and Gynecology, University of Alberta, Edmonton, Canada; 2 Department of Physiology, University of Alberta, Edmonton, Canada; 3 Women and Children’s Health Research Institute, University of Alberta, Edmonton, Canada; 4 Department of Molecular Pathophysiology, Shinshu University School of Medicine, Matsumoto, Japan; 5 Department of Life Innovation, Institute for Biomedical Sciences, Shinshu University, Matsumoto, Japan; 6 School of Pharmacy, University of Reading, Reading, UK; 7 Nuffield Department of Obstetrics and Gynaecology, University of Oxford, Oxford, UK

**Keywords:** Reproductive biology, Experimental models of disease

## Abstract

Vascular complications in pregnancy (e.g. preeclampsia) are a major source of maternal and foetal morbidity and mortality, and may be due to excessive release of placental syncytiotrophoblast-derived extracellular vesicles (STBEVs) into the maternal circulation. Increased activity of the multi-ligand scavenger receptor Lectin-like Oxidized LDL Receptor-1 (LOX-1) is associated with vascular dysfunction, and LOX-1 has been shown to interact with angiotensin II receptor type 1 (AT1). We hypothesized that STBEVs contribute to vascular dysfunction via LOX-1 and AT1 receptors during pregnancy. Uterine arteries from late pregnant wildtype and LOX-1 overexpressing mice were incubated overnight with or without STBEVs and vascular function was assessed using wire myography. STBEV-incubation decreased angiotensin II responsiveness only in wildtype mice, which coincided with decreased AT1 contribution and expression. Thus, STBEVs reduced angiotensin II responsiveness in normal pregnancy, but not in conditions of increased LOX-1 expression, suggesting that STBEVs (via LOX-1) play a role in normal adaptations to pregnancy. Oxidized LDL (a LOX-1 ligand) increased angiotensin II-induced vasoconstriction in STBEV-incubated arteries from both mouse strains, suggesting that the LOX-1 pathway may be involved in complicated pregnancies with elevated STBEVs and oxidized LDL levels (such as preeclampsia). These data increase our understanding of vascular complications during pregnancy.

## Introduction

Pregnancy is associated with many maternal physiological adaptations to pregnancy, which facilitate a major increase in cardiac output and blood flow, thereby supplying enough oxygen and nutrients to the developing foetus. Vascular adaptations to pregnancy include a refractory response to angiotensin II (Ang II)^1^ and a reduction in peripheral vascular resistance and blood pressure in the first half of pregnancy. Failure of some of these maternal pregnancy adaptations is associated with numerous pregnancy complications, such as intrauterine growth restriction and/or preeclampsia. These pregnancy complications are multifactorial and heterogenous conditions, therefore we have previously reported a potential converging pathway for the development of vascular dysfunction during pregnancy via the lectin-like oxidized LDL receptor-1 (LOX-1)^2–7^. For instance, many of the placental circulating factors that have been shown to be able to increase LOX-1 expression, such as cytokines like TNF-α and IFN-γ or oxidized LDL (oxLDL)(reviewed in^8^), are also associated with vascular dysfunction in women with the pregnancy complication preeclampsia^3,9^.

LOX-1 is a multi-ligand scavenger receptor that has been reported to contribute to vascular dysfunction in many cardiovascular diseases such as atherosclerosis, diabetes and hypertension^10–12^. In conditions of oxidative stress, oxidation of circulating LDL particles takes place leading to the formation of oxLDL, which is one of the main ligands for LOX-1^13^. OxLDL is extremely reactive and increased oxLDL levels can damage endothelial and immune cell function via LOX-1^12^. In the case of atherosclerosis, increased formation of oxLDL leads to dysfunction of endothelial cells, smooth muscle cells and macrophages (foam cell formation). Interestingly, pregnancy complications such as preeclampsia have also been associated with endothelial dysfunction and placental and vascular oxidative stress^14^. Some reports have shown that oxLDL levels are higher in women with preeclampsia and have been associated with an increased risk for developing preeclampsia^15,16^. Moreover, LOX-1 expression was shown to be increased in omental arteries from women with preeclampsia, as well as in aortas from pregnant rats with reduced perfusion pressure, suggesting a potential role for LOX-1 in complicated pregnancies^4,5^. Thus, increased LOX-1 expression together with increased oxLDL levels may play a role in the development of vascular/endothelial dysfunction in pregnancy.

In addition to oxLDL, many other LOX-1 ligands have been described such as activated platelets, apoptotic cells and even bacteria^12^. With LOX-1 being a multi-ligand scavenger receptor, we previously proposed that during gestation LOX-1 could be activated by circulating factors released from the placenta, such as syncytiotrophoblast extracellular vesicles (STBEVs)^6,7^. STBEVs are a heterogenous mixture of extracellular vesicles directly released into the maternal circulation from the foetal syncytiotrophoblast cells at the feto-maternal interface in the placenta^17^. STBEVs have been previously suggested to have a role in complicated pregnancies, as they were shown to activate endothelial and immune cells *in vitro* and potentially affect *ex vivo* vascular function^18–22^. Aligned with these studies, we previously showed that STBEVs reduced endothelium-dependent vasodilation in rat uterine arteries and, notably, that this was LOX-1 mediated^6^. Moreover, we showed that STBEVs induced peroxynitrite formation in cultured human umbilical vein endothelial cells, which was reduced by LOX-1 inhibition^7^.

Interestingly, intracellular signalling pathways of the LOX-1 receptor and the angiotensin II type I receptor (AT1) have been shown to be interconnected^23^, with LOX-1 signalling being dependent on the presence and activity of AT1 and vice versa^24^. This is of particular interest when considering the decreased sensitivity to Ang II during pregnancy and increased LOX-1 activity in vascular dysfunction. We have previously shown that Ang II responses were affected by STBEVs in uterine arteries from wildtype mice, but not in uterine arteries from LOX-1 knockout mice, suggesting a relationship between LOX-1, STBEVs and Ang II signalling^7^. However, the impact of increased LOX-1 expression, as has been observed in pathophysiological states (e.g. preeclampsia), on vascular dysfunction in pregnancy is not known. We hypothesized that STBEVs impair vascular function during pregnancy, specifically in conditions of increased LOX-1 expression or activation.

## Results

### Endothelium dependent vasodilation is more nitric oxide dependent by both LOX-1 overexpression and STBEV incubation

**Figure 1:**
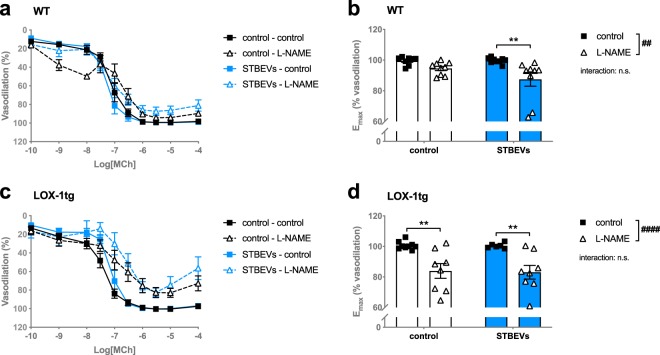
Nitric oxide contribution to endothelium-dependent vasodilation in uterine arteries from WT and LOX-1tg mice. (**a**,**c**) Contribution of nitric oxide to endothelium-dependent vasodilation responses to increasing doses of methylcholine (MCh) in uterine arteries from WT (**a**,**b**) and LOX-1tg mice (**c**,**d**) incubated overnight with (blue lines) or without STBEVs (black lines). Nitric oxide contribution to vasodilation was assessed by pre-incubation with (dashed lines; triangles) or without (solid lines; squares) the pan nitric oxide synthase inhibitor L-NAME. **(b**,**d)** Summary graphs show maximal vasodilation responses to MCh (E_max_) in uterine arteries from WT (**b**) and LOX-1tg mice (**d**) incubated overnight with (blue bars) or without STBEVs (white bars). Means with SEM; two-way ANOVA with Sidak’s multiple comparisons post-test; n.s.: not significant; ^##^p < 0.01, ^####^p < 0.0001, overall effect of treatment (control vs. L-NAME) in two-way ANOVA; **p < 0.01, control vs. L-NAME in multiple comparisons post-test. n = 8–10/group.

Endothelium-dependent vasodilation to MCh was not different in uterine arteries from WT versus LOX-1tg mice, with or without STBEVs (pEC_50_ (mean ± SEM): WT: 7.11 ± 0.09; WT + STBEVs: 7.28 ± 0.10; LOX-1tg: 7.37 ± 0.09; LOX-1tg +STBEVs: 7.15 ± 0.09). However, when arteries were pre-incubated with L-NAME to assess nitric oxide contribution to vasodilation, there was a significant contribution of nitric oxide in the STBEV-incubated arteries in WT mice (decreased E_max_: Fig. 1a,b), while L-NAME incubation did not change the maximum vasodilation response in the WT arteries without STBEVs. Moreover, uterine arteries from LOX-1tg mice, both with and without STBEV incubation, showed contribution of nitric oxide to endothelium-dependent vasodilation (Fig. 1c,d). Uterine artery endothelial nitric oxide synthase (eNOS) expression was not different in arteries from WT compared to LOX-1tg mice and was not affected by STBEV-incubation (WT + control: 14.3 ± 1.7 a.u.; WT + STBEVs: 12.2 ± 1.9 a.u.; LOX-1tg +control: 12.5 ± 2.2 a.u.; LOX-1tg+STBEVs: 12.9 ± 1.8 a.u.).

### Activation of LOX-1 by oxLDL impaired endothelium-mediated vasodilation responses

**Figure 2:**
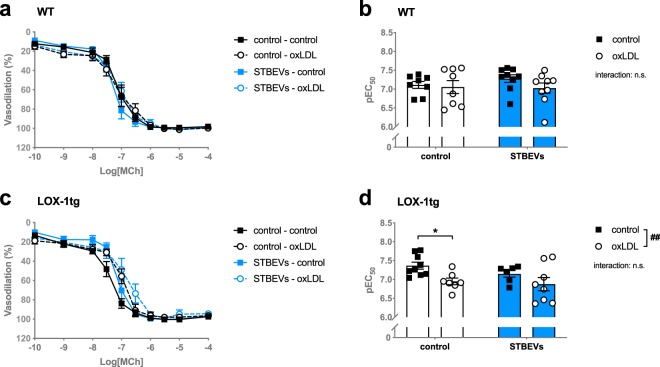
Effect of oxLDL on endothelium-dependent vasodilation in uterine arteries from WT and LOX-1tg mice. (**a**,**c**) Contribution of pre-incubation with (dashed lines; circles) or without (solid lines; squares) oxLDL (oxLDL-induced LOX-1 activation; 10 minutes before the start of the curve) to endothelium-dependent vasodilation responses to increasing doses of methylcholine (MCh) in uterine arteries from WT (**a**,**b**) and LOX-1tg mice (**c**,**d**) incubated overnight with (blue lines) or without STBEVs (black lines). **(b**,**d)** Summary graphs show the dose of to MCh necessary to establish 50% of total relaxation (pEC_50_) in uterine arteries from WT (**b**) and LOX-1tg mice (**d**) incubated overnight with (blue bars) or without STBEVs (white bars). Means with SEM; two-way ANOVA with Sidak’s multiple comparisons post-test; n.s.: not significant; ^##^p < 0.01, overall effect of treatment (control vs. oxLDL) in two-way ANOVA; *p < 0.05, control vs. oxLDL in multiple comparisons post-test. n = 8–9/group.

Pre-incubation with the LOX-1 ligand oxLDL did not alter endothelium-dependent vasodilation responses to MCh in WT mice (Fig. 2a,b). However, in uterine arteries from LOX-1tg mice, there was an overall decrease in sensitivity to MCh (i.e. decreased pEC_50_) after oxLDL stimulation (Fig. 2c,d). Moreover, this effect was mainly due to a significant reduction in MCh sensitivity in control vessels from LOX-1tg mice after oxLDL stimulation, while this was not the case in STBEV-incubated arteries (Fig. 2c,d).

### STBEVs decrease Ang II responses in WT, but not LOX-1tg mice

**Figure 3:**
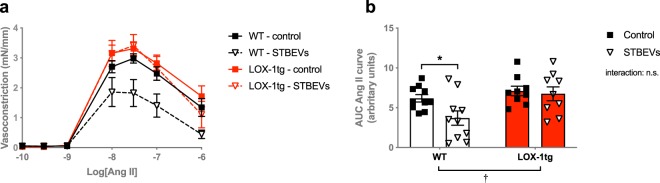
Ang II-induced vasoconstriction responses in uterine arteries from WT and LOX-1tg mice. (**a**) Vascular responses to increasing doses of Ang II in uterine arteries from WT (black lines) and LOX-1tg mice (red lines) incubated overnight with (dashed lines; triangles) or without STBEVs (solid lines; squares). **(b)** Summary graph show area under the curve (AUC) in uterine arteries from WT (white bars) and LOX-1tg mice (red bars) incubated overnight with (triangles) or without STBEVs (squares). Means with SEM; two-way ANOVA with Sidak’s multiple comparisons post-test; n.s.: not significant; ^†^p < 0.05 overall effect of strain (WT vs. LOX-1tg) in two-way ANOVA; *p < 0.05, control vs. STBEVs in multiple comparisons post-test. n = 9–10/group.

**Figure 4:**
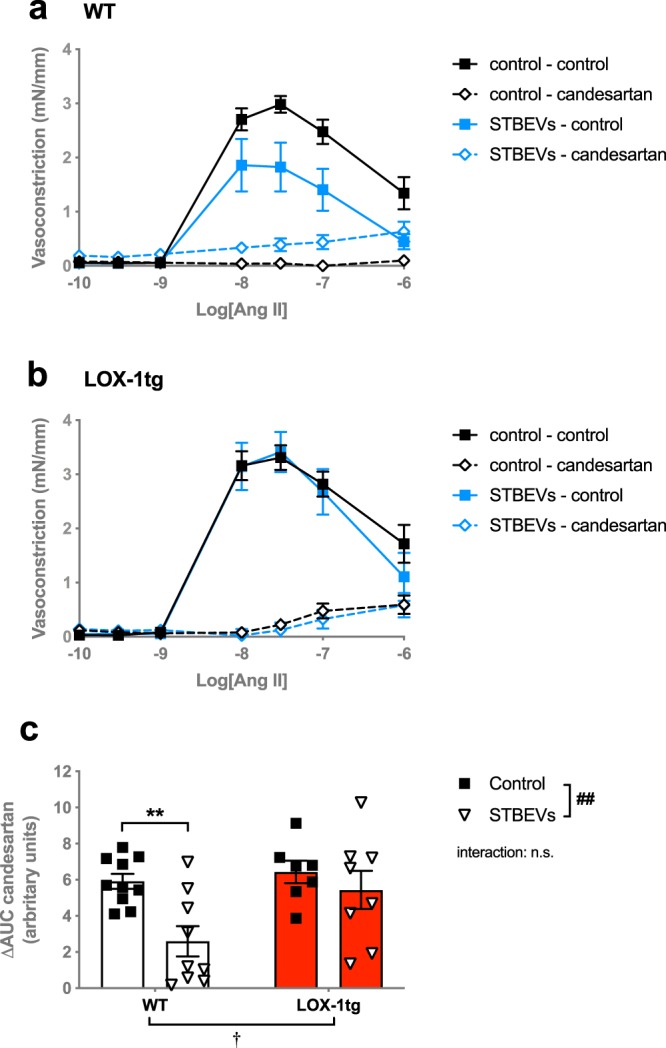
AT1 receptor contribution to Ang II-induced vasoconstriction responses in uterine arteries from WT and LOX-1tg mice. (**a**,**b**) The contribution of the AT1 receptor on the Ang II-induced vasoconstriction responses in uterine arteries from WT (**a**) and LOX-1tg mice (**b**) incubated overnight with (blue lines) or without STBEVs (black lines) was assessed by pre-incubation with (dashed lines; diamonds) or without (solid lines; squares) the AT1 receptor antagonist candesartan. **(c)** Summary graph shows the delta area under the curve (ΔAUC) between the control and candesartan-incubated uterine arteries from WT (white bars) and LOX-1tg mice (red bars) incubated overnight with (triangles) or without STBEVs (squares). Means with SEM; two-way ANOVA with Sidak’s multiple comparisons post-test; n.s.: not significant; ^†^p < 0.05 overall effect of strain (WT vs. LOX-1tg); ^##^p < 0.01, overall effect of treatment (control vs. STBEVs) in two-way ANOVA; **p < 0.01, control vs. STBEVs in multiple comparisons post-test. n = 7–10/group.

Contractile responses to Ang II were similar in uterine arteries from WT and LOX-1tg mice (Fig. 3a,b). In the presence of STBEVs, there was a decreased responsiveness to Ang II in the uterine arteries from WT mice (Fig. 3a,b). In contrast to our hypothesis, this decreased Ang II response to STBEVs was not observed in arteries from LOX-1tg (Fig. 3a,b). Pre-incubation with candesartan (an AT1 receptor antagonist) significantly inhibited contractile responses to Ang II in all groups (Fig. 4a,b). The decrease in constriction (i.e. the AT1 receptor contribution calculated as delta AUC) was greater in uterine arteries from LOX-1tg compared to WT mice (Fig. 4c). Moreover, in arteries from WT mice (but not LOX-1tg mice), the AT1 receptor contribution (delta AUC) was significantly decreased by STBEVs (Fig. 4c).

**Figure 5:**
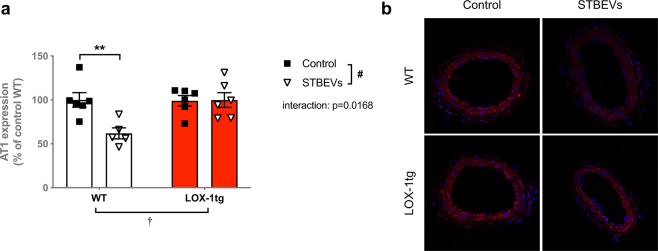
Uterine artery AT1 receptor expression. (**a**) Using immunofluorescent staining, AT1 receptor expression was assessed in cryo-sections of uterine arteries from WT (white bars) and LOX-1tg mice (red bars) incubated overnight with (triangles) or without STBEVs (squares). **(b)** Representative uterine artery images for each of the groups (red = AT1 and blue=nuclear dapi staining). Means with SEM; two-way ANOVA with Sidak’s multiple comparisons post-test; ^#^p < 0.05, overall effect of treatment (control vs. STBEVs) in two-way ANOVA; ^†^p < 0.05, overall effect of strain (WT vs. LOX-1tg) in two-way ANOVA; **p < 0.01, control vs. STBEVs in multiple comparisons post-test. n = 5–6/group.

In line with the reduced Ang II responsiveness after STBEV incubation in uterine arteries from WT mice, uterine artery AT1 receptor expression was decreased (about 40%) by STBEV-incubation in the WT, but not in the LOX-1tg mice (significant interaction; Fig. 5a,b).

### oxLDL increased Ang II responses in STBEV-incubated arteries

**Figure 6:**
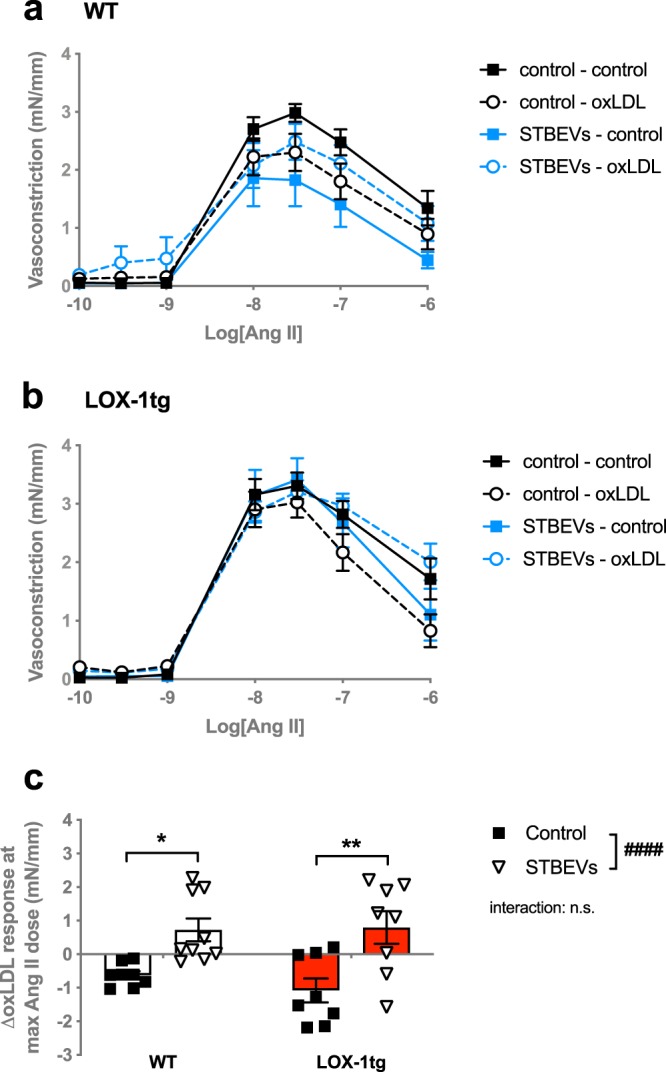
Effect of oxLDL on Ang II-induced vasoconstriction responses. (**a**,**b**) Contribution of pre-incubation with (dashed lines; circles) or without (solid lines; squares) oxLDL (10 minutes before the start of the curve) to Ang II-induced vasoconstriction responses in uterine arteries from WT (**a**) and LOX-1tg mice (**b**) incubated overnight with (blue lines) or without STBEVs (black lines). **(c)** Summary graph shows delta (Δ) response to Ang II at the highest Ang II dose between the control and oxLDL-incubated uterine arteries from WT (white bars) and LOX-1tg mice (red bars) incubated overnight with (triangles) or without STBEVs (squares). Means with SEM; two-way ANOVA with Sidak’s multiple comparisons post-test; n.s.: not significant; ^####^p < 0.0001, overall effect of treatment (control vs. STBEVs) in two-way ANOVA; *p < 0.05, **p < 0.01, control vs. STBEVs in multiple comparisons post-test. n = 8–9/group.

Pre-incubation with oxLDL (i.e. LOX-1 activation) decreased Ang II responsiveness in control uterine arteries from both WT and LOX-1tg mice (Fig. 6a,b). However, in uterine arteries pre-incubated with STBEVs, oxLDL increased Ang II responsiveness in both WT and LOX-1tg mice compared with control arteries (Fig. 6c).

## Discussion

In the current study, we showed that increased expression of LOX-1 during pregnancy increased uterine artery nitric oxide contribution to vasodilation but also resulted in impaired endothelium-dependent relaxation in the presence of oxLDL and STBEVs. Moreover, we showed that incubation with STBEVs reduced responsiveness to Ang II in only WT arteries, which appeared to be mediated by a decreased AT1 receptor expression. We also showed an interaction between STBEV-incubation and the response to oxLDL. Our data suggest that both STBEVs and LOX-1 may play important roles in vascular adaptations to normal pregnancy. Interestingly, these adaptations in normal pregnancy were absent in LOX-1tg mice, thus implicating that LOX-1 contributes to vascular dysfunction in complicated pregnancies associated with increased LOX-1 expression.

STBEVs are released during normal pregnancy and in increasing amounts at the end of gestation, as the placenta grows and releases more debris into the maternal circulation^25^. Several studies have reported higher amounts of STBEVs in women with preeclampsia^26^, and increased expression of proinflammatory factors on the STBEVs^27^, which led to the hypothesis that STBEVs could contribute to the development of vascular dysfunction in this disorder^17^. In general, extracellular vesicles and exosomes were shown to play a role in many biological processes, such as intercellular communication^28,29^. However, what role STBEVs during normal gestation could play is still unknown, as they have been regarded more as placental ‘garbage bags’ than essential messengers. Excitingly, our data show a potential physiological role for STBEVs during normal pregnancy, by contributing to a reduction in vascular Ang II sensitivity, which is an essential pregnancy adaptation. There is limited knowledge on how the refractoriness to Ang II develops, making our data of particular interest. Moreover, as previously suggested, our data confirm that having a higher expression of LOX-1 during pregnancy, whether that is due to a predisposition or established due to inflammatory factors or other circulating factors (potentially STBEVs themselves) could be detrimental to vascular function during gestation. Therefore, increased LOX-1 expression together with increased circulating levels of factors such as oxLDL and STBEVs are likely to be risk factors for the development of vascular complications during pregnancy, such as preeclampsia.

In the seminal study by Gant *et al*. in 1973, responsiveness to Ang II was decreased in normal pregnant women compared to women with pregnancy-induced hypertension who responded to Ang II similar to women in the nonpregnant state^1^. However, the mechanisms for this have not been elucidated, especially since Ang II levels are lower in women with preeclampsia compared to control pregnant women (reviewed in^30^). Remarkably, and unexpectedly, our data showed that STBEV-incubation significantly reduced Ang II responsiveness in uterine arteries from WT mice, which coincided with a reduction in AT1 receptor expression. Thus our data suggest that STBEVs could play a role in the reduced Ang II responsiveness during normal pregnancy, via decreasing AT1 receptor expression. Even more interesting, this reduction in Ang II response by STBEVs was completely absent in the LOX-1 overexpressing mice. Thus, having increased expression of LOX-1 in these arteries reduced the capacity for STBEVs to decrease AT1 receptor expression, thereby affecting Ang II responsiveness. Several studies have reported that the LOX-1 receptor and the AT1 receptor interact allosterically and are both activated by stimulation of either LOX-1 or AT1^23,24^. Our data support these findings and suggest that basal LOX-1 expression allows for an interaction with AT1 in a way so that STBEV-incubation decreases AT1 expression. These data align with the concept that activation of AT1 results in internalization of the receptor and thus tachyphylaxis (reduced responsiveness to a ligand) and the fact that this was not shown in the LOX-1tg arteries, suggests that this STBEV-effect was LOX-1 mediated. However, the exact mechanism remains elusive, and further studies are needed.

Alternatively, we have previously shown that both LOX-1 and platelet-activating factor (PAF) receptors were increasingly involved in vascular function in the Reduced Uterine Perfusion Pressure (RUPP) model, where activation of PAF receptors, which can induce vasodilation, compensated for the activation of LOX-1^31^. Hence, it may be suggested that STBEVs potentially also behave as PAF-like lipids and activate PAF receptors. This could induce vasodilation in WT arteries (and thus reduce Ang II responsiveness), while in LOX-1tg arteries the balance between LOX-1 and PAF activation is shifted by increased LOX-1 activation, thereby reducing the PAF-mediated effects.

Interestingly, the overexpression of LOX-1 in our transgenic LOX-1tg mice is linked to the preproendothelin-1 promotor, hence LOX-1 is primarily overexpressed at sites of endothelin-1 production, i.e. cardiac tissues and endothelial cells^32^. Endogenous LOX-1 is expressed on smooth muscle cells but primarily on vascular endothelial cells^12^. In contrast, even though AT1 (and AT2) receptors are expressed on endothelial cells^33^, they are mainly expressed on the vascular smooth muscle cells. This raises the question as to how endothelial LOX-1 could interact with/influence smooth muscle contractions to Ang II in the vascular smooth muscle cells. As the whole segment of uterine artery was incubated with STBEVs, STBEVs could have activated LOX-1 receptors on both endothelial and smooth muscle cells. It would be expected that the decreased Ang II responsiveness is primarily a smooth muscle cell-mediated effect. However, when LOX-1 is overly expressed, pathological intracellular pathways may be activated, for instance via production of reactive oxygen species in endothelial cells^34^. Moreover, endothelial modulation of Ang II responses via AT1 receptors on endothelial cells has been described, yet the data are conflicting. Some studies have reported increased production of nitric oxide and prostaglandins by endothelial AT1 activation^33^ (reviewed in^35^), which is interesting as interplay between nitric oxide and Ang II has been suggested (reviewed in^36^) and nitric oxide has been shown to downregulate AT1 receptor expression in vascular smooth muscle cells^37^. Conversely, many others have shown the opposite, where endothelial AT1 activation decreased nitric oxide and eNOS expression^38^ (reviewed in^39^), and with many of these studies performed *in vitro*, the *in vivo* or *ex vivo* role of endothelial modulation of Ang II responses requires more information.

Ang II responsiveness was decreased after LOX-1 activation by oxLDL in uterine arteries from both WT and LOX-1tg mice, suggesting that, even though some effects of STBEVs are mediated via LOX-1 (as observed in the endothelium-dependent vasodilation data), this likely is not the only signalling mechanism via which STBEVs affect vascular function. Indeed, it has been suggested that, just like other extracellular vesicles, STBEVs could potentially activate other scavenger receptors^40^. Interestingly, oxLDL had more effect on endothelium-dependent vasodilation compared to the effect on Ang II responses that are mainly AT1 and vascular smooth muscle cell-mediated, which aligns with LOX-1 being an endothelial receptor and highlights the complexity of the LOX-1/AT1 association on different vascular cell types. In both WT and LOX-1 overexpressing arteries, LOX-1 activation by oxLDL decreased Ang II responsiveness in control arteries, while Ang II responsiveness was increased in the STBEV-incubated arteries. This suggests that having both increased levels of circulating oxLDL and STBEVs could worsen the effect of either of these factors alone and, together with our endothelium-dependent vasodilation data, this is in line with the general hypothesis that vascular dysfunction in women with preeclampsia (i.e. conditions of increased LOX-1 expression) is multifactorial and heterogenous^3^. In agreement with our data, Yamamoto *et al*. reported that oxLDL disturbed endothelium-dependent vasodilation in aortas, and this was not the case in arteries from AT1 knockout mice^23^. Therefore this cross-reactivity and interaction between LOX-1 and AT1 may also be contributing to other vasoreactivity, such as vascular responses to other vasoconstrictors, but this remains to be investigated.

In contrast to our data showing that vasoconstriction responses to Ang II were impaired by STBEVs, we observed that endothelium-dependent vasodilation responses were not different in uterine arteries from WT or LOX-1tg mice, nor was there an effect of STBEV-incubation. We have previously observed similar results in uterine arteries from WT and LOX-1 deficient mice^7^, however, in our previous study in rats we found STBEVs decreased maximum endothelium-dependent vasodilation responses^6^. Moreover, in human tissue, Cockell *et al*. found that STBEVs decreased endothelium-dependent vasodilation responses in vessels from subcutaneous fat^18^, while Van Wijk *et al*. did not observe any differences after STBEV-exposure in myometrial arteries^21^, suggesting that there are both species- and vascular bed-related differences in the effect of STBEVs.

Compared to controls, the effect of both L-NAME or oxLDL on vasodilation responses was more pronounced in uterine arteries with increased LOX-1 expression compared to WT arteries. In contrast, even though the overall responsiveness to MCh was unchanged by STBEV-incubation, the contribution of nitric oxide to vasodilation was increased by STBEVs in uterine arteries from WT mice. Interestingly, similar dichotomous effects of low versus higher LOX-1 activity (i.e. the STBEV or oxLDL-incubated samples or LOX-1 overexpressing arteries in our experiments) have been previously described by Akhmedov *et al*.^34^. They showed that increased LOX-1 activation by high oxLDL levels resulted in activation of different intracellular pathways that promotes ROS production and NF-kB activity^34^. However, a basal amount of endothelial LOX-1 activation by low levels of circulating oxLDL activates protective intracellular signalling pathways by SIRT1 activation and inhibition of NF-kB activity, thereby having a physiologically important function of removing harmful oxLDL particles from our circulation^34^. In line with our previous data in rats^6^, our data thus show that STBEVs affect the mechanisms of endothelium-dependent vasodilation (i.e. nitric oxide contribution) via LOX-1 during pregnancy. Our functional data using a pan-nitric oxide synthase inhibitor suggest an increased NOS activity in uterine arteries from LOX-1tg mice and by STBEV incubation. Although eNOS expression was not different between the groups, there could be enhanced activation of eNOS (i.e. increased phosphorylation). Moreover, as L-NAME is a pan-NOS inhibitor, there may be other isoforms (i.e. iNOS or nNOS) involved as well. Thus, alterations in activity of specific NOS isoforms remains to be confirmed.

Alternatively, our data may also suggest activation of alternative receptors by STBEVs, or direct non-receptor mediated uptake of STBEVs. Being such a heterogenous population of extracellular vesicles of which different pathways for cellular uptake have been described^40^, it is unlikely that LOX-1 would be the only mechanism via which STBEVs could be affecting endothelial function. Moreover, although interesting, our study did not distinguish between larger microvesicles or exosomes because it is unknown what type/size of extracellular vesicle could potentially activate LOX-1, hence this remains to be further investigated. Although beyond the scope of our current study, studies analysing a dose-response curve to STBEVs, as well as assessing the effect is EV-mediated (i.e. using proteolytic enzymes to degrade the particles), will be part of our future directions.

We also assessed the effect of stimulation with the LOX-1 ligand oxLDL on vascular function. OxLDL incubation decreased the sensitivity to methylcholine in uterine arteries from LOX-1tg mice, but not in the WT arteries. This is in line with previous data assessing the effect of oxLDL on vascular function in rat, mouse and human arteries^2,14,23,31^, and confirms that the overexpression of LOX-1 in the LOX-1tg mice is functional. Aside from being a well-known ligand for LOX-1, oxLDL is also a circulating factor that is increased in complicated pregnancies, such as preeclampsia^15,16^. Our data confirm our hypothesis that specifically in conditions of increased LOX-1 expression oxLDL reduces endothelium-dependent vasodilation and may thus contribute to systemic vascular dysfunction that is observed in women with preeclampsia. Of interest was that the STBEV-incubated LOX-1tg arteries did not significantly respond to oxLDL as the control arteries did. This suggests that both oxLDL and STBEVs work via similar mechanisms: via the LOX-1 receptor.

In summary, interestingly and unexpectedly, our data suggest a role for placental-derived STBEVs and LOX-1 in the normal vascular adaptations to pregnancy. Specifically, the uterine artery in this milieu may benefit notably from having a refractory response to vasoconstrictors such as Ang II, thereby enabling the sufficient supply of blood transporting oxygen and nutrients to the developing foetus. From a pathophysiological perspective, however, STBEVs could have detrimental effects in women that have pre-existing (potentially subclinical) disease associated with increased vascular LOX-1 expression, such as in hypertension, diabetes, atherosclerosis or dyslipidaemia^10–12^. Alternatively or concurrently, other circulating factors released by the placenta that have been shown to increase LOX-1 expression^8^ have also been associated with vascular dysfunction in women with preeclampsia^3,9^, which supports our hypothesis of LOX-1 as a converging factor for vascular dysfunction during pregnancy.

## Methods

### Ethics statement

All animal experiments were approved by the Alberta Health Sciences Animal Policy and Welfare Committee of the University of Alberta, Canada (AUP #242) in accordance with the Canadian Council on Animal Care Guidelines. Ethics for the collection of STBEVs from human placentas were approved by the Oxfordshire Research Ethics Committee C, were carried out in accordance with the Declaration of Helsinki, and written informed consent was obtained from the patient.

### Syncytiotrophoblast extracellular vesicle collection

STBEVs were collected as described previously by Dr. Tannetta at the University of Oxford^41^. In short, an intact lobule of a placenta obtained from a normal pregnant patient undergoing an elective caesarean section was perfused using perfusion media (20 µm filtered after preparation, details in^41^) for 3 hours. Larger cells and aggregates were removed from the maternal side perfusion media by centrifugation (1500 g for two times) and the supernatant was collected. STBEVs (containing both microparticles and exosomes) were collected by ultracentrifugation at 150,000 g, after which the pellet was washed with filtered (20 µm) phosphate buffered saline (PBS) once (150,000 g) and the pellet was reconstituted into filtered PBS and frozen (4.95 mg/mL protein). As previously described, the sample was checked for placental origin (placental alkaline phosphatatse, PLAP + ) and size distribution by nanoparticle tracking analysis and flow cytometry^41,42^. STBEVs were prepared using established methodologies that have consistently yielded preparations of vesicles with high purity and low contamination with blood cell derived vesicles. Extensive details of the procedure and isolation and characterization of the STBEVs (in accordance with the MISEV guidelines^43^) can be found in^41,42,44–46^. After collection, STBEV samples were frozen at −80 °C and transported to the University of Alberta for the *ex vivo* vascular function experiments.

### Experimental setup animal experiments

Transgenic LOX-1 overexpressing mice (LOX-1tg; on C57BL/6 background) were obtained from Dr. Sawamura in Japan and were generated by inserting bovine LOX-1 downstream of the preproendothelin-1 promotor, as described in more detail by Inoue *et al*.^32^ This bovine LOX-1 is functional, as shown by increased human oxLDL uptake in heart vessels^32^. Female WT (+/+) and LOX-1tg mice (tg/0) (aged 7–12 weeks; not litter mates) were impregnated by overnight housing with a WT male. The next day mice were deemed pregnant by plug testing, which was designated as gestational day (GD) 0.5. On GD18.5, pregnant mice were sacrificed by exsanguination while under isoflurane anaesthesia, and uterine tissues were obtained. Uterine arteries were immediately isolated (main branch uterine artery) from the uterine tissue, cut into 2 mm segments, mounted on a single 25 µm wire and incubated overnight in microfuge tubes on a rocking platform at 4 °C with either 200 µg/ml STBEVs in physiological salt solution (PSS) or as a control artery in PSS only, as previously described^6,7^. Vascular function was assessed the next morning using wire myography.

### Vascular function using myography

The next morning, vascular function was assessed by mounting the 2 mm segments of uterine artery onto a wire myograph system (DMT, Copenhagen, Denmark), using 25 µm wires. Any remaining 2 mm segments (depending on the original size of the isolated uterine artery) were snap frozen in OCT (optimal cutting temperature) compound (Sakura Finetek, Torrance, CA, USA) for immunofluorescent staining. After mounting, vessels were allowed to equilibrate until stable and were then normalized (IC100 = 0.8; kPa=7.32). After normalization the vessels were allowed to equilibrate for 20 minutes, after which they received the first wake up dose of 1×10^−5^ mol/L phenylephrine (Sigma-Aldrich) for 5 minutes. After washing with PSS thrice and 10 minutes rest the vessels received a second wake up dose of phenylephrine (1×10^−5^ mol/L), followed by a dose (3×10^−6^ mol/L) of the endothelium-dependent vasodilator methylcholine (MCh; Sigma-Aldrich) to test for endothelial intactness and function. After washing three times with PSS, the arteries were equilibrated for 10 minutes. Arteries were then pre-incubated either 1) without inhibitors, 2) with the pan nitric oxide synthase inhibitor N(G)-Nitro-L-arginine methyl ester (L-NAME; 1×10^−4^ mol/L, Sigma-Aldrich) for 30 minutes or 3) with the LOX-1 receptor ligand oxidized LDL (50 µg/mL, medium oxidized LDL, Kalen Biomedical, Germantown, MD, USA) for 10 minutes prior to pre-constriction of all vessels using the EC80 dose of phenylephrine (3×10^−6^ mol/L). After reaching plateau constriction to phenylephrine (5 minutes) endothelium-dependent vasodilation responses were assessed with a vasodilation cumulative concentration response curve to MCh (CCRC; 1×10^−10^ to 1×10^−4^ mol/L MCh; doses were added in 2 minute intervals). Vessels were then washed four times using PSS, equilibrated for 10 minutes, and pre-incubated either 1) without inhibitors, 2) with AT1 receptor antagonist candesartan (1×10^−9^ mol/L, Selleck Chemicals/Cedarlane, Burlington, ON, Canada) for 30 minutes, or 3) with the LOX-1 receptor ligand oxidized LDL (50 µg/mL) for 10 minutes prior to starting the Ang II CCRC (1×10^−11^ to 1×10^−6^ mol/L Ang II (Sigma-Aldrich); subsequent doses were added in 2 minute intervals if maximum constriction/plateau to the respective dose was reached). All vessels were washed four times with PSS and each bath was incubated with a high potassium salt solution (KPSS; 123 mmol/L) for 5 minutes or until plateau, to assess non-receptor-mediated vasoconstriction responses. All data (maximum relaxation responses to MCh and maximum constriction to Ang II and KPSS) were analysed using LabChart software (ADInstruments; Colorado Springs, USA).

### Assessment of AT1 and eNOS expression in uterine arteries using immunofluorescent staining

Uterine artery cryostat sections (8 µm) were warmed up to room temperature, fixed in ice-cold acetone (−20 °C) for 10 minutes and allowed to dry. Slides were washed with PBS (pH 7.5) thrice for 5 minutes and incubated with blocking solution (2% bovine serum albumin [BSA] in PBS supplemented with 5% normal donkey serum for AT-1 and 2%BSA in PBS for eNOS) for 60 minutes at room temperature to inhibit non-specific binding. After aspiration of the blocking solution, sections were incubated overnight at 4 °C in a humid chamber with primary antibodies diluted in 2% BSA/PBS against the AT1 receptor (1:500; rabbit-anti-AGTR-1, NOVUS Biologicals) or against eNOS (1:200; rabbit-anti-eNOS/NOS3; Santa Cruz Biotechnologies, Dallas, TX, US). The next day, sections were washed with PBS thrice for 5 minutes, excess liquid was removed and incubated with secondary antibody diluted in 2% BSA/PBS (1:250 donkey-anti-rabbit-Alexa Fluor 546 for AT-1; goat-α-rabbit-AF546 for eNOS; Thermo Fisher Scientific, Waltham, MA, USA) for 60 minutes at room temperature in a humid chamber in the dark. Sections were washed thrice with PBS (5 minutes), covered using mounting medium containing DAPI (Vector Laboratories, Burlingame, CA, USA), left to dry overnight and images were obtained the next day.

### Fluorescent images using confocal microscopy and image analysis

Images of fluorescent staining of eNOS and AT1 were obtained using a confocal Zeiss LSM 700 microscope and Zen Black software (Zeiss, Toronto, ON, Canada). Gains for AF546 (red) and DAPI (blue) were set using the blank control sections (incubated with secondary antibodies only, without primary antibodies). Images were analysed using ImageJ software (U. S. National Institutes of Health, Bethesda, Maryland, USA)^47^. The mean fluorescent intensity (MFI) was calculated after selecting the entire vessel area (endothelial plus smooth muscle layer). Artefacts (folds or nonspecific staining such as particles) were excluded from the analysis. If several images were obtained (depending on the number of uterine artery segments that were snap frozen in OCT after uterine artery isolation) the mean value for those images was taken.

### Statistical analysis

Data were analysed using Graphpad Prism 8 (Graphpad Software, San Diego, US). A two-way ANOVA with Sidak’s multiple comparisons test was used to assess the effect of strain (WT versus LOX-1tg) and treatment (control versus STBEVs) on the summary EC_50_, AUC, or E_max_ values, depending on the most appropriate option to summarize the vascular response data. Statistical differences were regarded significant when p < 0.05.
